# Comprehensive analysis of TCP transcription factors and their expression during cotton (*Gossypium arboreum*) fiber early development

**DOI:** 10.1038/srep21535

**Published:** 2016-02-09

**Authors:** Jun Ma, Fang Liu, Qinglian Wang, Kunbo Wang, Don C. Jones, Baohong Zhang

**Affiliations:** 1 Department of Biology, East Carolina University, Greenville, NC 27858, USA; 2 State Laboratory of Cotton Biology, Institute of Cotton Research, Chinese Academy of Agricultural Sciences, Anyang, Henan 455000, P. R. China; 3 Henan Institute of Sciences and Technology, Xinxiang, Henan 453003, P. R. China; 4 Cotton Incorporated, Cary, NC 27513, USA

## Abstract

TCP proteins are plant-specific transcription factors implicated to perform a variety of physiological functions during plant growth and development. In the current study, we performed for the first time the comprehensive analysis of TCP gene family in a diploid cotton species, *Gossypium arboreum*, including phylogenetic analysis, chromosome location, gene duplication status, gene structure and conserved motif analysis, as well as expression profiles in fiber at different developmental stages. Our results showed that *G. arboreum* contains 36 TCP genes, distributing across all of the thirteen chromosomes. GaTCPs within the same subclade of the phylogenetic tree shared similar exon/intron organization and motif composition. In addition, both segmental duplication and whole-genome duplication contributed significantly to the expansion of GaTCPs. Many these TCP transcription factor genes are specifically expressed in cotton fiber during different developmental stages, including cotton fiber initiation and early development. This suggests that TCP genes may play important roles in cotton fiber development.

TCP proteins constitute a family of plant-specific transcription factors widely distributed in angiosperms^1^,^2^. The TCP gene family was termed after its founding members: TEOSINTE BRANCHED 1 in *Zea mays*, CYCLOIDEA in *Antirrhinum majus* and PCF in *Oryza sativa*^1^. Since its initial identification and characterization in 1999, the TCP family has become one of the focuses of plant studies due to its importance in the evolution and developmental control of plant form^2^. TCP proteins are defined by a 59-residue-long basic helix-loop-helix (bHLH) structure called TCP domain, which provides this family with the ability to bind GC-rich DNA sequence motifs^2^. According to the secondary structure prediction, the basic region of TCP domain is followed by two helices separated by a loop^2^. In addition, phylogenetic analysis showed that TCP proteins can be classified into two subfamilies based on their DNA binding domain structure^2^. To date, more than 20 TCP family members have been identified in a number of monocot and eudicot plants, such as Arabidopsis^3^, *Oryza sativa*^4^, *Vitis vinifera* and *Populus trichocarpa*^5^.

In plants, the TCP transcription factor family has been implicated to perform a variety of physiological functions during plant growth and development, such as branching, regulation of the circadian clock, seed germination, gametophyte development, hormone pathways, leaf development, mitochondrial biogenesis, flower development and cell cycle regulation^2^,^6^,^7^,^8^,^9^,^10^,^11^,^12^. Recently, evidences indicated that TCP proteins also play a significant role in fiber development^13^,^14^, which makes it necessary to identify and characterize TCP family members in cotton, one of the most important economic crops and natural fiber sources all over the world^15^.

Cotton comprises both diploid and tetraploid species, belonging to the *Gossypium* genus. The most commonly cultivated cotton species for fiber and oil production is upland cotton (*Gossypium hirsutum*), an AD tetraploid evolved from A-genome diploids such as *G. arboreum* and D-genome diploids like *G. raimondii* at around 1–2 million years ago^16^. Up to now, only two TCP family members have been functionally characterized in cotton, suggesting that TCP genes may play key roles in fiber development^13^,^14^. Therefore, there is an urgent need to perform a genome wide analysis of this family in cotton. The recent completion of the sequencing of *G. arboreum* genome allowed us to characterize all cotton TCP genes.

In the current study, we performed for the first time the comprehensive analysis of TCP gene family in *G. arboreum*, including phylogenetic analysis, chromosome location, gene duplication status, gene structure and conserved motif analysis, as well as tissue specific expression profiles. Our findings will lay solid foundation to better understand the function and evolutionary history of GaTCPs, and will help further investigation of the detailed molecular and biological functions of TCP members in cotton.

## Results

### Identification of the TCP gene family in *G. arboretum*

The TCP transcription factor family is featured by a highly conserved TCP domain at the N-terminus. To identify this family in *G. arboretum*, the profile hidden Markov Models of TCP domain (PF03634) downloaded from Pfam was used as query to run Hmmsearch against the *G. arboretum* genome. As a result, 58 putative TCP genes were identified. After the removal of 22 redundant sequences based on multiple sequence alignment, 36 candidate TCP genes sequences were manually inspected with ScanProsite to confirm the existence of the conserved TCP domain. As expected, they all contained the TCP domain, suggesting that they were members of the TCP gene family. The 36 TCP genes were further named as *GaTCP1* to *GaTCP25* according to *Arabidopsis* TCP nomenclature suggestions. Table 1 summarized their gene symbols, corresponding gene names, sequence length, molecular weights, isoelectric points and chromosome location.

### Evolutionary analysis of the TCP transcription factor family

**Figure 1:**
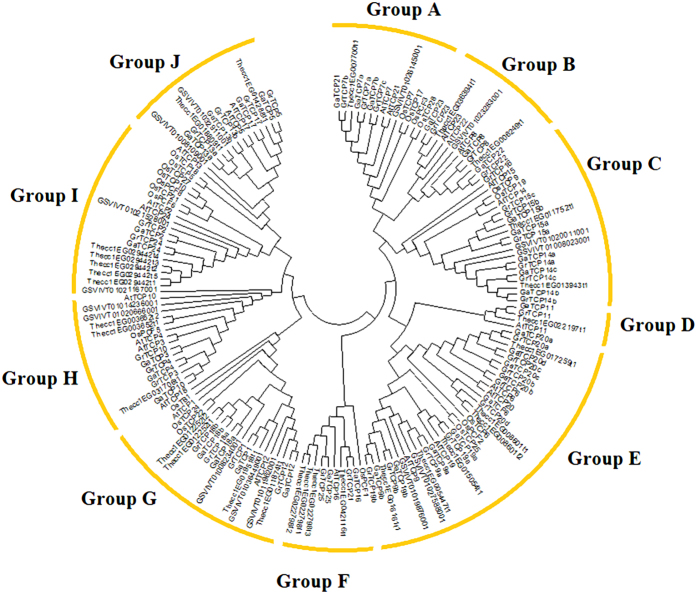
Phylogenetic tree of TCP proteins from *Gossypium arboreum*, *G. raimondii*, *Theobroma cacao*, *Vitis vinifera*, *Arabidopsis thaliana* and *Oryza sativa*. The phylogenetic tree was generated using the Neighbor-Joining (NJ) method implemented in the MEGA 6.0 software with JTT model and pairwise gap deletion option. The bootstrap analysis was conducted with 1000 iterations.

In order to explore the evolutionary relationships of the TCP transcription factor family, an unrooted phylogenetic tree was generated using the full length TCP protein sequences from *G. arboreum*, *G. raimondii*, *Theobroma cacao*, *Vitis vinifera*, *Arabidopsis thaliana* and *Oryza sativa*. As illustrated in the Neiboring-Joining phylogenetic tree (Fig. 1), the TCP transcription factor family was classified into ten distinct subgroups designated as Group A to Group J. Group E is composed of 33 members, constituting the largest clade among all subgroups, while Group G is the second largest clade, consisting of 21 TCP protein sequences. The smallest clade is Group D, which contains only four TCP proteins. Generally speaking, all subgroups contain at least five plant species except Group D, exhibiting an interspersed distribution. This may imply that the divergence of these species took place after the TCP transcription factor family expanded. Noticeable, the TCPs from *G. arboretum* and *G. raimondii* formed quite a few clusters of homologs in all subfamilies due to their high sequence similarity and close evolutionary relationship that the divergence of the two cotton species occurred only 2–13 million years ago^17^. In addition, the cotton TCPs (GrTCPs and GaTCPs) were more closely allied to TCP proteins from *T. cacao* than from other plant species, consistent with the fact that *G. arboreum*, *G. raimondii* and *T. cacao* originated from a common ancestor 18–58 million years ago^17^. Based on multiple sequence alignments, orthologous TCP gene pairs between T. cacao and G. arboreum were identified: GaTCP14b/Thecc1EG013943t1, GaTCP15b/Thecc1EG011752t1, GaTCP11/Thecc1EG022197t1, GaTCP23/Thecc1EG036394t1, GaTCP22/Thecc1EG006249t1, GaTCP19a/Thecc1EG015054t1, GaTCP20a/Thecc1EG017259t1, GaTCP16/Thecc1EG042116t1, GaTCP12/Thecc1EG011874t1, and GaTCP1/Thecc1EG019518t1. Moreover, Group B and Group D did not contain *O. sativa* TCP proteins, suggesting that the TCP family members in the two subgroups were either lost in *O. sativa* or obtained after the divergence of monocots and eudicots. Additionally, the phylogenetic analysis also showed that the TCP members from different plant species were not evenly distributed in some subgroups. GaTCPs, for example, were overrepresented than AtTCPs in Group C and Group E, in which GaTCPs were over two times the number of AtTCPs. This may indicate that the TCPs went through differential expansion in *G. arboreum* and in *Arabidopsis*.

### Chromosomal distribution and gene duplication

**Figure 2:**
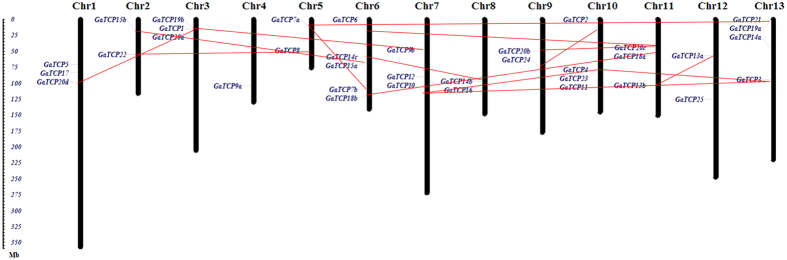
Chromosomal location and gene duplication status of TCP genes from *Gossypium arboreum* on 13 chromosomes. The scale represents megabases (Mb). The chromosome numbers are indicated above each vertical bar. The red lines connect the duplicated gene pairs.

The 36 *G. arboretum* genes were mapped onto chromosomes in order to elucidate their chromosomal distribution and gene duplication status. As shown in Fig. 2, the 36 GaTCPs were scattered throughout all 13 chromosomes of *G. arboretum*. Chromosome 6 had the highest number of five TCP genes, followed by Chromosome 10 and Chromosome 13 with four TCP genes. The lowest number of GaTCPs were observed in Chromosome 4 with one TCP gene. In general, the TCP genes were more evenly distribute across *G. arboretum* chromosomes than that in *G. raimondii*^18^.

In addition, the gene duplication events were further investigated to reveal the expansion mechanism of the TCP gene family in *G. arboretum*. The criteria described in previous studies were employed to identify paralogous genes pairs. As a result, 15 pairs of putative paralogous TCP genes were found in *G. arboretum* with high gene and protein sequence identity and similarity, accounting for about 70% of the whole GaTCP gene family. As illustrated in Fig. 2, all of the gene pairs were distributed on different chromosomes, while no tandem duplication events could be observed, suggesting that segmental duplications contributed a lot to the amplification of the GaTCP gene family. Additionally, in order to gain more insights of the evolutionary history of the GaTCP gene family, the DnaSP program was used to calculate the approximate dates of duplication events, dating the duplication events of GaTCPs between 11.28 Mya (million years ago) to 36.51 Mya, with an average of 19.7 Mya. The detailed analysis for the duplicated gene pairs was listed in Table 2.

### Gene structure and conserved motifs

**Figure 3:**
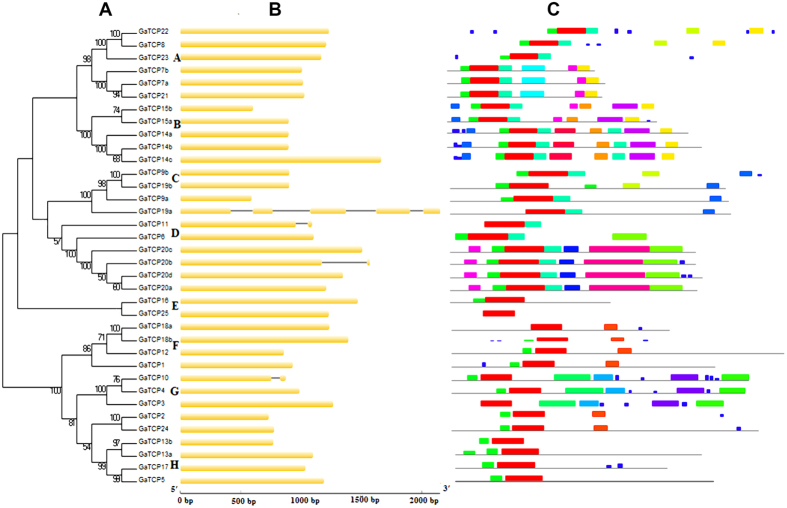
Phylogenetic analysis, exon/intron organization and motif composition s of *Gossypium arboreum* TCP genes. (**A**) The phylogenetic tree was generated using the Neighbor-Joining (NJ) method implemented in the MEGA 6.0 software with JTT model and pairwise gap deletion option. The bootstrap analysis was conducted with 1000 iterations. (**B**) The exon/intron distribution of *G. arboreum* TCP genes. Exons and introns are represented by green boxes and black lines, respectively. (**C**) The motif compositions of *G. arboreum* TCP genes. Each color represents a specific motif.

To get a better understanding of the diversification of the TCP genes in *G. arboretum*, the exon/intron organization and conserved motifs of GaTCPs were analyzed. A new Neiboring-Joining phylogenetic tree was constructed using the protein sequences of GaTCPs, dividing the TCP family into eight subclades. As shown in Fig. 3, over 80% of GaTCP genes were intronless, which was quite similar to the structure of *G. raimondii* TCP genes^18^. Generally speaking, most GaTCPs within the same subclades exhibited similar gene structure in terms of numbers and lengths of introns and exons. Subclade A and H, for instance, contained intronless genes with similar exon lengths. In contrast, great structure variants were observed in Subclade C. In addition, the MEME programs was used to predict motif composition, identifying twenty conserved motifs in GaTCPs. Subsequently, the program InterProScan was employed to annotate these motifs. The results showed that the only motif that hit for the database was the conserved TCP domain (the red motif), which was found in all GaTCPs. Moreover, similar to the exon/intron organization, members belonging to the same subclades also showed similar motif composition, indicating their functional similarities. Additionally, some motifs were only presented at specific subclades, suggesting that they may perform subclade specific functions.

### Expression profiles of cotton TCP genes at different developmental stages

**Figure 4:**
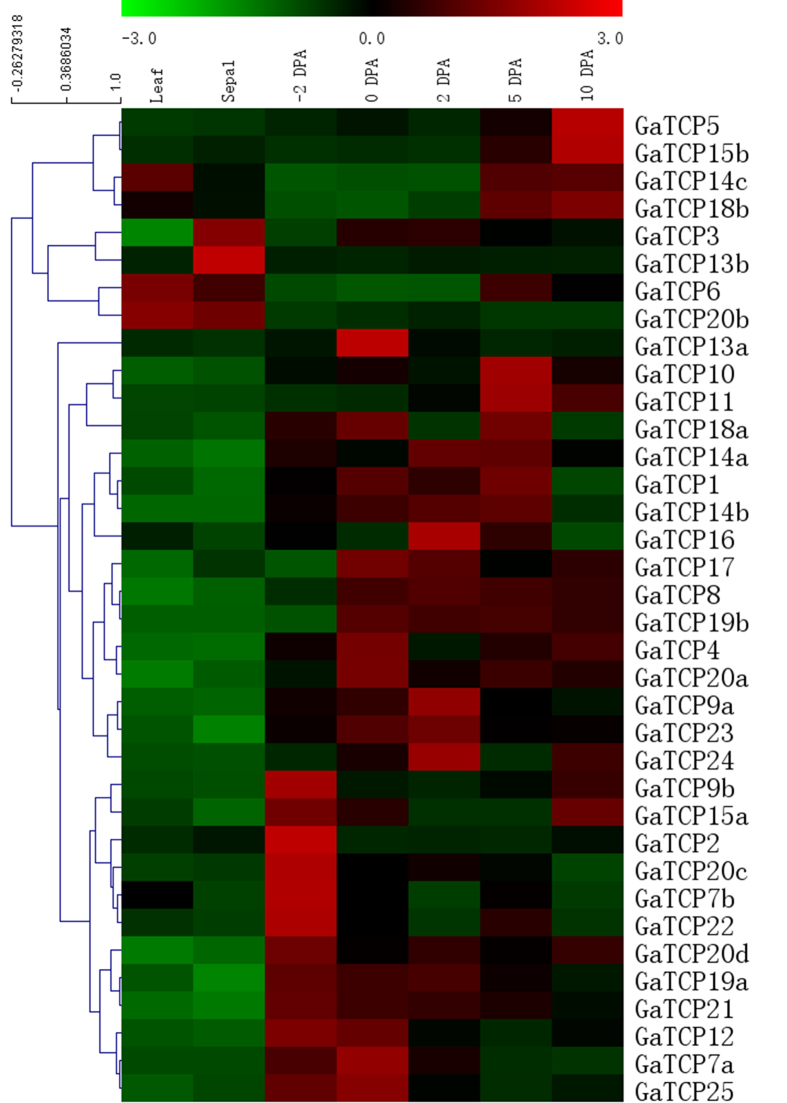
Expression profiles of *G. arboreum* TCP genes in different tissues and at different fiber developmental stage. The expression levels are represented by the color bar.

**Figure 5:**
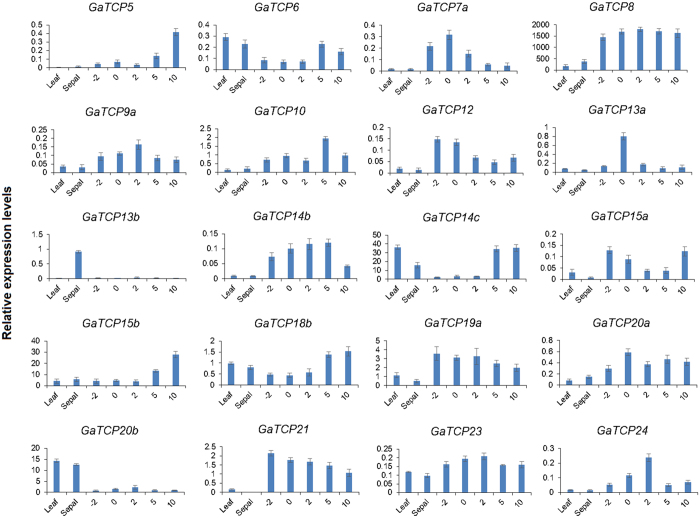
Expression profiles of 20 *G. arboreum* TCP genes in different tissues and at different fiber developmental stage. The X-axis represents different tissues or developmental stages while the Y-axis represents relative expression levels against the reference gene *SAD1*. Error bars are drawn based on standard deviation for three replicates.

In order to shed light on the potential physiological functions of the TCP gene family in *G. arboretum* at different developmental stages as well as in cotton fiber development, their expression profiles were investigated using Real-Time Quantitative Reverse Transcription PCR on several different organs, including leaves, sepals and fibers at −2, 0, 2, 5 and 10 DPA. As shown in Figs 4 and 5, the majority of the TCP genes exhibited diverse expression profiles, while a few of them showed similar expression patterns. For example, a number of genes, including GaTCP5, GaTCP8, GaTCP12, GaTCP13a, GaTCP14b, GaTCP15a, GaTCP15b, GaTCP19a, GaTCP21, were exclusively highly expressed in fiber, while GaTCP13b and GaTCP20b were preferentially expressed in leaves or sepals at the high levels. Additionally, several TCP genes had high expression levels in all the tissues examined, such as GaTCP6, GaTCP18b and GaTCP23. Among those genes that were highly expressed in fibers, GaTCP7a, GaTCP12, GaTCP13a and GaTCP24 were highly expressed at the fiber initiation stage (from −2 to 2 DPA), whereas the expression levels of GaTCP5, GaTCP10, GaTCP14c and GaTCP15b were significantly high at the fiber elongation stage (from 5 to 10 DPA). Remarkably, GaTCP8 had extremely high expression levels at all the fiber developmental stages tested.

## Discussion

The TCP transcription factor family plays important roles in many biological processes during plant growth and development, such as fiber development^13^,^14^, seed germination^19^,^20^, leaf development^21^, hormone signal transduction^22^ and flower development^21^,^22^,^23^. The recent availability of *G. arboreum* genome sequences^17^ allowed us to perform a comprehensive analysis of this family in cotton, including phylogenetic analysis, chromosome location, gene duplication status, gene structure and conserved motif analysis, as well as tissue specific expression profiles.

### Evolutionary conservation and divergence of the TCP gene family in cotton

The *G. arboreum* genome contains almost the same number of TCP genes as in *G. raimondii*^18^ and have more than 50% more than in *Arabidopsis*^2^, which is in consistency with the number of protein coding genes in each species^17^,^24^,^25^. It has been reported that *Arabidopsis* and *G. arboreum* evolved from a common ancestor at around 93 Mya and subsequently underwent paleopolyploidy events^24^,^26^. Our phylogenetic analysis showed that TCP genes in cotton and *Arabidopsis* might go through differential expansion caused by gene duplication, an important source of raw genetic materials to the evolution of complex plant systems^27^,^28^. This is supported by the fact that the number of paralogous TCP gene pairs accounted for over 70% of the entire TCP gene family in *G. arboreum* and that segmental duplication is a predominant duplication event for GaTCPs. Previous studies indicated that gene duplication contributed to the amplification of gene family members on various scales, such as tandem duplication, segmental duplication and whole-genome duplication, and that the expansion of regulatory genes can hardly ever be achieved simply through single gene duplication alone^27^,^28^,^29^, implying that genome duplication may also contribute to the amplification of the GaTCP gene family. According to a recent study, a recent and an ancient whole genome duplication event have occurred in *G. arboreum* at approximately 13–20 and 115–146 Mya, respectively^17^. Our results indicated that the average duplication date of GaTCPs was around 19.7 Mya, which is consistent with the recent whole genome duplication event. This suggests that genome duplication may also play a significant role in the expansion of the GaTCP gene family.

In addition to gene duplication status, differences in exon/intron organizations can also shed light on the evolutionary history of gene families. In this study, we compared the gene structure of GaTCPs with their homologous counterparts in *Arabidopsis*^18^. The results showed that eight of ten pairs of TCP genes shared conserved exon/intron distribution in terms of exon length and intron numbers, whereas two pairs displayed some extent of divergence. AtTCP12, for instance, contained one more intron than its counterpart GaTCP12. Such intron loss of gain may result from insertion/deletion events in the process of evolution^30^.

According to previous reports, upland cotton (*G. hirsutum*), an AD tetraploid, evolved from A-genome diploids *G. arboreum* and D-genome diploids *G. raimondii* at around 1–2 Mya^16^. Due to high similarities between the A and D genomes in terms of gene sequence and genome organization, the TCP family members in *G. arboreum* and *G. raimondii* formed a lot of clusters of homologs in the phylogenetic tree and shared almost identical exon/intron structures as well as motif compositions^18^. Our phylogenetic analysis also showed that GaTCPs and GrTCPs were more closely allied to TCP proteins from *T. cacao*, a close relative of cotton in the *Malvaceae* family, than from other plant species, consistent with the fact that *G. arboreum*, *G. raimondii* and *T. cacao* originated from a common ancestor 33 Mya^17^. Since GaTCPs and GrTCPs duplicated at around 19.7 million years ago, these duplications happened after their divergence from *T. cacao* and *Arabidopsis* but before the reunion of the A and D genome diploids that gave rise to upload cotton.

### Functional divergence of the TCP gene family in cotton

It has been widely recognized that duplicated genes undergo one of the following evolutionary fates: pseudogenization (in which one copy becomes unexpressed or functionless), conservation of gene function (in which both copies maintain the same function), subfunctionalization (in which the ancestral function is subdivided between copies), and neofunctionalization (in which one copy acquires a new function)^27^. In the present study, the expression profiles of GaTCPs at different developmental stages were investigated to reveal their functional divergence during plant growth and development.

Our results showed that the majority of the paralogous GaTCP gene pairs exhibited differential expression profiles. GaTCP7a, for example, was preferentially expressed in fiber at the initiation stage, while its paralogous counterpart GaTCP23 was highly expressed in fiber at both the initiation stage and the elongation stage. GaTCP14c had extremely high expression level in leaf and fiber at the elongation stage, whereas GaTCP14b was relatively highly expressed in fiber at the initiation stage. In general, the expression patterns of GaTCP genes imply that the TCP family may perform multiple physiological functions in *G. arboreum*, especially in fiber initiation and elongation. Remarkably, some of these findings have already been experimentally confirmed through analysis of mutant cotton species with reduced and/or overexpressed TCP activities. For instance, the expression level of GaTCP15b was significantly high in fiber at the elongation stage. Hao *et al*. demonstrated that GbTCP, the homologous counterpart of GaTCP15b in *G. barbadense*, confers cotton fiber elongation by regulating JA biosynthesis and response and other pathways using RNAi silencing technique^14^. In addition, Wang *et al*. demonstrated that the GhTCP14 from upland cotton functions as a crucial regulator in auxin-mediated elongation of cotton fiber cells^13^, which is in agreement with our result that GaTCP14c was highly expressed in fiber at the elongation stage. However, further functional analysis of GaTCPs are still needed in order to determine which evolutionary fates the duplicated GaTCP genes undergo during the process of sequence and functional evolution.

### Comparison of the TCP gene family between *G. arboreum* and *G. raimondii*

Molecular systematics studies show that the most cultivated cotton species *G. hirsutum* is produced by interspecific hybridization of A-genome (like *G. arboreum*) and D-genome (like *G. raimondii*) diploid progenitor species, which makes it necessary to make a comparison analysis on gene structure, chromosomal distribution and gene duplication of the TCP gene family between *G. arboreum* and *G. raimondii*. Based on genome scans of the two cotton species, a total of 38 and 36 TCP genes were identified in *G. raimondii* and *G. arboreum*, respectively. Comparison of gene structures indicated that the orthologous TCP gene pairs exhibited a highly conserved distribution of exons and introns either in terms of intron numbers or gene length. In addition, these TCP genes also shared similar gene duplication status that both segmental duplication and whole-genome duplication contributed significantly to the expansion of TCP genes. In contrast, great variety was observed in their chromosomal distribution. For instance, The GrTCPs were unevenly distributed across 11 out of the 13 *G. raimondii* chromosomes, ranging widely from 0 to 8 genes per chromosome, whereas the GaTCPs were more evenly scattered throughout all 13 chromosomes of *G. arboreum*. The cause of this great variety is still unclear. It might be related to the high Long Terminal Repat (LTR) activities that contributed to the twofold increase in the size of the *G. arboreum* genome.

## Materials and Methods

### Identification of TCP genes and proteins

The genome sequence of *G. arboreum* was downloaded from the Cotton Genome Project (CGP) (http://cgp.genomics.org.cn/). To identify the TCP family in *G. arboretum*, the profile hidden Markov Models of TCP domain (PF03634) downloaded from Pfam was used as query to run Hmmsearch against the *G. arboretum* genome (P-value = 0.0011). The candidate TCP genes were further aligned to remove redundant sequences^31^. Subsequently, the TCP sequences were manually inspected with ScanProsite to confirm the presence of the conserved TCP domain^32^. The TCP gene and protein sequences from *Theobroma cacao*, *Vitis vinifera*, *Arabidopsis thaliana* and *Oryza sativa* were retrieved from PlantTFDB plant transcription factor database, while the GrTCP sequences were obtained from previous studies^18^.

### Phylogenetic analysis

Cluster X program was employed to perform multiple sequence alignments with default parameters^33^. Unrooted phylogenetic trees were subsequently constructed using the Neighbor-Joining (NJ) method implemented in the MEGA 6.0 software with JTT model and pairwise gap deletion option^34^. The bootstrap analysis was conducted with 1000 iterations.

### Chromosomal location and gene duplication

The physical location data of GaTCP genes were retrieved from *G. arboreum* genome. Mapping of these GaTCP genes was then performed using MapInspect software. Gene duplication was defined according to the criteria descripted in previous studies: the aligned region of two sequences covers over 70% of the longer sequence and the similarity of the aligned region is over 70%^35^,^36^. In addition, the DnaSp software^37^ was employed to calculate Ka (nonsynonymous substitution rate) and Ks (synonymous substitution rate), which was further used to estimate the date of duplication events with the formula T = Ks/2λ, assuming clock-like rate (λ) of 1.5 synonymous substitutions per 10^8^ years for cotton^24^.

### Gene structure and conserved motif

The exon/intron organizations of GaTCPs were inferred through comparison of genomic sequences and CDS sequences in the gene structure display server^38^. The program MEME^39^ was employed to identify conserved motifs in GaTCPs with the following parameters: the optimum width of motif, 6–250; the maximum number of motif, 20; the number of repetitions, any. In addition, motif annotation was performed using the program InterProScan^40^.

### RNA isolation and Real-time quantitative RT-PCR analysis

Total RNA was isolated from *G. arboreum* leaves, sepals and fibers at −2, 0, 2, 5 and 10 days post anthesis (DPA) using the mirVanaTM miRNA Isolation Kit (Ambion, USA). Subsequently, the NanoDrop ND-1000 Spectrophotometer was employed to determine RNA concentration and quality. cDNA was then synthesized from 1 μg of total RNA with poly-T primers using the TaqMan® MicroRNA Reverse Transcription Kit (Applied Biosystems, USA). RT-qPCR was later conducted on 7300 Real-Time PCR System (Applied Biosystems, USA) according to the manufacture’s protocol. The amplification parameters were as follows: enzyme activation at 95 °C for 10 min, 45 cycles of denaturation at 95 °C for 15 s and annealing/elongation at 60 °C for 60 s. The relative expression levels was calculated according to previous studies^18^. A reference genes *SAD1* was used to normalize the expression values. There were three biological replicates and each biological replicate was run three times. Finally, the software MultiExperiment Viewer was used to construct heatmap representation for expression patterns.

## Additional Information

**How to cite this article**: Ma, J. *et al*. Comprehensive analysis of TCP transcription factors and their expression during cotton (*Gossypium arboreum*) fiber early development. *Sci. Rep.* **6**, 21535; doi: 10.1038/srep21535 (2016).

## Figures and Tables

**Table 1:** TCP gene family in *G. arboretum.*

Gene Name	Gene symbol	Length (aa)	MW (Da)	pI	Chr. Location
GaTCP1	Cotton_A_09911	397	43545.24	9.21	chr3:16012758:16014321
GaTCP2	Cotton_A_26168	410	44917.18	6.77	chr10:15878745:15879977
GaTCP3	Cotton_A_23161	409	44273.72	6.78	chr13:94068971:94070200
GaTCP4	Cotton_A_22289	443	48760.48	6.06	chr10:78860246:78861667
GaTCP5	Cotton_A_31971	325	36033.79	5.8	chr1:70185179:70186156
GaTCP6	Cotton_A_23025	250	26502.48	9.58	chr6:20222965:20223732
GaTCP7a	Cotton_A_08973	258	26739.99	9.72	chr5:10164781:10165557
GaTCP7b	Cotton_A_14593	243	25308.51	9.99	chr6:109156974:109157705
GaTCP8	Cotton_A_24144	486	50969.89	7.36	chr5:48534877:48536337
GaTCP9a	Cotton_A_10947	338	35365.29	9.08	chr4:104148560:104149576
GaTCP9b	Cotton_A_14431	385	41254.45	8.95	chr7:47526308:47527465
GaTCP10	Cotton_A_20110	448	48746.25	6.6	chr7:116121419:116122765
GaTCP11	Cotton_A_24059	200	21687.42	8.32	chr10:109535667:109536269
GaTCP12	Cotton_A_37122	501	55859.83	6.82	chr7:89657212:89658717
GaTCP13a	Cotton_A_27227	309	34245.51	8.71	chr12:56165133:56166062
GaTCP13b	Cotton_A_14726	285	31976.83	7.94	chr11:102879533:102880390
GaTCP14a	Cotton_A_09220	395	42218.14	6.91	chr13:44785981:44787168
GaTCP14b	Cotton_A_02703	418	44468.80	8.60	chr8:96754543:96755799
GaTCP14c	Cotton_A_27685	406	43980.41	6.88	chr6:58440407:58441627
GaTCP15a	Cotton_A_06142	342	37377.38	8.53	chr6:68046188:68047216
GaTCP15b	Cotton_A_33342	365	39684.16	9.54	chr2:18664013:18665110
GaTCP16	Cotton_A_10509	196	21078.67	8.56	chr8:103306077:103306667
GaTCP17	Cotton_A_19125	266	30312.08	7.78	chr1:83153095:83153967
GaTCP18a	Cotton_A_07573	329	37748.89	9.02	chr11:53845238:53846327
GaTCP18b	Cotton_A_01394	367	41500.33	9.08	chr6:120385396:120386499
GaTCP19a	Cotton_A_21588	341	36882.22	6.26	chr13:4529921:4530946
GaTCP19b	Cotton_A_09964	335	35753.32	9.42	chr3:14665842:14666849
GaTCP20a	Cotton_A_40823	300	32008.71	7.93	chr3:18568141:18569043
GaTCP20b	Cotton_A_07501	298	31710.22	7.28	chr9:49546327:49547223
GaTCP20c	Cotton_A_39272	298	31420.10	9.64	chr11:43972643:43973539
GaTCP20d	Cotton_A_22689	306	32794.32	9.27	chr1:99325941:99326861
GaTCP21	Cotton_A_26482	255	26370.49	9.66	chr13:2155968:2156735
GaTCP22	Cotton_A_27060	553	58300.67	6.73	chr2:54926878:54928539
GaTCP23	Cotton_A_03998	418	44401.83	6.72	chr10:80131534:80132790
GaTCP24	Cotton_A_02913	463	50231.92	7.01	chr9:73463153:73464544
GaTCP25	Cotton_A_37650	431	47737.38	6.47	chr12:125507830:125509978

**Table 2:** Dates of duplication for the duplicated gene pairs.

Gene 1	Gene 2	ks	T = Ks/2λ
GaTCP2	GaTCP24	0.3869	12.89667
GaTCP3	GaTCP10	0.3868	12.89333
GaTCP10	GaTCP4	0.4783	15.94333
GaTCP7a	GaTCP7b	0.5899	19.66333
GaTCP9b	GaTCP19b	0.4529	15.09667
GaTCP13b	GaTCP13a	0.6527	21.75667
GaTCP14b	GaTCP14c	0.5637	18.79
GaTCP15a	GaTCP15b	0.6751	22.50333
GaTCP17	GaTCP5	1.0293	34.31
GaTCP18a	GaTCP18b	1.0954	36.51333
GaTCP20a	GaTCP20d	0.4212	14.04
GaTCP20c	GaTCP6	0.3384	11.28
GaTCP20b	GaTCP20a	0.4097	13.65667
GaTCP20d	GaTCP20b	0.4599	15.33
GaTCP22	GaTCP8	0.9276	30.92
